# Activation-induced deaminase (AID) localizes to the nucleus in brief pulses

**DOI:** 10.1371/journal.pgen.1007968

**Published:** 2019-02-27

**Authors:** Quy Le, Nancy Maizels

**Affiliations:** 1 Molecular and Cellular Biology Program, University of Washington, Seattle, Washington, United States of America; 2 Departments of Immunology and Biochemistry, University of Washington, Seattle, Washington, United States of America; Stanford University School of Medicine, UNITED STATES

## Abstract

Activation-induced deaminase (AID) converts C to U and 5-methyl-C to T. These mutagenic activities are critical to immunoglobulin (Ig) gene diversification and epigenetic reprogramming, but they must be tightly controlled to prevent compromising cell fitness. AID acts in the nucleus but localizes predominately to the cytoplasm. To address this apparent paradox, we have carried out time-lapse imaging of AID in single living B cells and fibroblasts. We demonstrate that AID enters the nucleus in brief (30 min) pulses, evident in about 10% of cells in the course of a single cell cycle (24 hr imaging). Pulses do not depend on AID catalytic activity, but they are coordinated with nuclear accumulation of P53. Pulsing may protect cells from pathologic consequences of excess exposure to AID, or enable AID to synchronize its activity with transcription of genes that are AID targets or with nuclear entry of factors that act at sites of AID-catalyzed DNA deamination to promote Ig gene diversification or epigenetic reprogramming.

## Author summary

Activation-induced deaminase (AID) is a mutagenic factor that plays a critical role in immunoglobulin gene diversification and also functions in early development to reprogram methylated regions of the genome. AID must be tightly regulated to prevent compromising cell fitness, but how this occurs is not thoroughly understood. One level of regulation is known to be nuclear entry and exit, but spatiotemporal regulation of AID had not been examined at the level of single cells. We have carried out time-lapse imaging of individual cells expressing fluorescent-tagged AID. Our movies show that AID enters the nucleus in brief pulses, of about 30 minutes duration. Pulses occur in only a fraction of cells in the course of one cell cycle and appear to respond to a metronome intrinsic to individual cells. AID pulses are coordinated with pulses of P53, which regulates the cellular response to DNA damage. Pulsing may enable AID to synchronize both with factors that respond to AID-initiated damage and with factors that regulate transcription of AID target genes.

## Introduction

Activation-induced deaminase (AID) is essential for the three processes that diversify immunoglobulin (Ig) gene sequence and structure: somatic hypermutation, class switch recombination, and gene conversion [1–6]. In B cells of the germinal center and other lymphoid tissues, AID deaminates C to U at the rearranged and transcribed Ig genes and factors from the base excision repair or mismatch repair pathways then remove the U, thereby creating a DNA nick that can initiate somatic hypermutation, gene conversion or class switch recombination [7, 8]. AID also erases methylation marks to reprogram transcription in very early development [9–12] and in germinal center B cells [13] by deaminating 5-meC to T, which is then replaced by C lacking a methylation mark.

Even though AID must act in the nucleus, it localizes primarily to the cytoplasm. Cytoplasmic localization of AID has been documented in the vast majority (>90%) of cells in a variety of tissues and cell populations, including fixed germinal center B cells that express endogenous AID and cultured cells of other lineages that express fluorescent tagged AID (e.g. [14–18]). AID can cause genomic instability or chromosomal translocations [19–23] and the cytoplasmic localization of AID protects cells from deleterious consequences of nuclear AID activity. AID is sequestered in the cytoplasm [24] and requires active transport to enter the nucleus [25]. AID nuclear persistence is limited both by ubiquitin-dependent proteolysis [26, 27] and export by the CRM1-dependent nuclear export pathway [15, 16, 28]. Nuclear AID is potentially toxic, and mutations that impair export or proteolysis of nuclear AID can compromise cell viability [22, 27, 29].

A great deal is known about AID regulation, but most current understanding derives from analyses of cell populations. Analyses of single living cells can provide new insights into regulation, as gene expression and protein activity and localization can vary dynamically among cells within a nominally homogeneous population [30]. We have therefore carried out live cell imaging of cells expressing AID tagged with fluorescent protein. Here, we demonstrate that AID enters the nucleus in brief pulses, of about 30 min duration. Pulses are observed in about 10% of cells in asynchronous culture over the course of 24 hr imaging. They are independent of AID catalytic activity. AID nuclear accumulation stimulates nuclear accumulation of P53, but while AID appears to be purged from the nucleus at the end of each pulse, nuclear P53 persists in cells that have pulsed. These results identify a new pathway of AID regulation.

## Results

### Live cell imaging reveals that AID pulses in the nucleus

**Fig 1:**
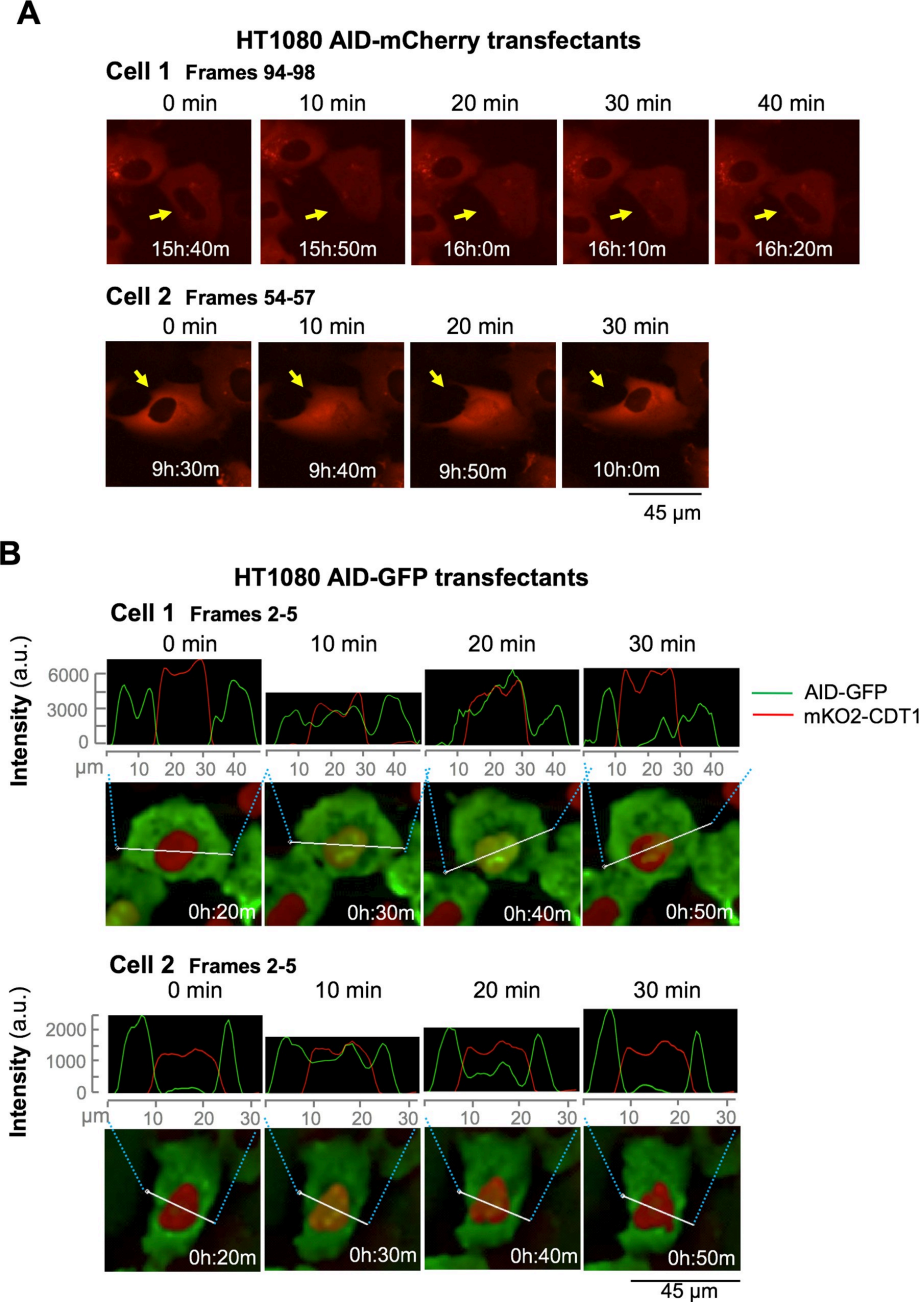
AID pulses in the nucleus. (A) Representative frames captured by live cell imaging, showing two HT1080 AID-mCherry transfectants at 10 min intervals. Arrows point to cells that pulse in still frames. Pulses captured in images shown occur in S1 Movie in a single cell at the center of frames 57–60 (upper images) and frames 71–74 (lower images). (B) Representative frames captured by live cell imaging, showing AID-GFP (green) and mKO2-CDT1 (red, nuclear) signals in HT1080 transfectants at 10 min intervals. Above, trace of red and green signal amplitudes across region indicated by bar drawn in image of cell. Pulses captured in images occur in S2 Movie, cell at center left of frames 2–5 (upper images); and S3 Movie, cell at lower center of frames 2–5 (lower images).

AID is expressed in B cells and in non-lymphoid cells. To facilitate imaging, we first examined AID-mCherry transfectants of human HT1080 cells, which derive from a fibrosarcoma and form stable attachments to the surface of a slide that maintain cell position during extended periods of imaging. Cells were imaged at 10 min intervals, using a DeltaVision microscope equipped with an environmental chamber to maintain the cells at 37°C, 5% CO_2_. Time-lapse imaging produced a striking result. The AID-mCherry signal was cytoplasmic in most cells, as predicted by a number of studies documenting cytoplasmic localization of AID; and dispersed throughout the cell during mitosis (S1 Fig, S1 Movie), as previously reported [6]. However, in a fraction of cells, AID-mCherry appeared to localize to the nucleus in brief pulses (S1 Movie), illustrated by two representative pulses of 40 (above) and 30 min (below) duration (Fig 1A).

We then analyzed AID-GFP transfectants, to confirm that results were not an artefact of the mCherry tag. In order to distinguish the nucleus and cytoplasm in these living cells, we examined HT1080 cells expressing both AID-GFP and the mKO2-CDT1 fusion protein, which confers a red signal to nucleus during G1 phase [31]. Like the AID-mCherry signal, the AID-GFP signal was cytoplasmic in most cells and appeared in brief pulses in the nuclei of a minority of cells. Consecutive time-lapse images of two representative cells at high resolution illustrate how the green AID-GFP signal is initially exclusively cytoplasmic (t = 0); then nuclear, overlapping the red nuclear mKO2-CDT1 signal (t = 10, 20 min); then cytoplasmic once again (t = 30 min; Fig 1B; S2 and S3 Movies).

To establish whether pulses occur in B cells as well as fibroblasts, we analyzed AID-mCherry transductants of the human B cell line, Ramos. Ramos cells derive from a Burkitt lymphoma and express endogenous AID, and they carry out ongoing somatic hypermutation of their endogenous V_H_ and V_L_ regions. The small size of B cells makes them very sensitive to radiation, so cells were imaged at 15 min rather than 10 min intervals, to help preserve viability. In Ramos AID-mCherry transductants, the AID-mCherry signal appeared in the nucleus in transient pulses like those observed in HT1080 transfectants expressing fluorescent-tagged AID (S2 Fig, S4 and S5 Movies). We conclude that AID localizes to the nucleus in short pulses in B cells as well as fibroblasts.

### AID nuclear pulses are asynchronous within a cell population and occur throughout cell cycle

Pulses have previously been shown to regulate nuclear access of about a dozen proteins in yeast, all of them transcription factors [32–34]; and five human proteins, including P53 [35] and NF-κB [36–38]. These factors all respond to specific signals that synchronize pulses among cells in a population. To determine if AID nuclear pulses occur in response to analogous cues, we carried out detailed characterization of pulsing in individual HT1080 AID-mCherry transfectants. By time-lapse imaging at 10 min intervals over the course of 24 hr, we recorded 289 individual cells (948 hr total imaging time). We observed one or more AID nuclear pulses in 36 cells (12.5%; S1 Table), but pulses were not synchronous among cells in a population. Thus, pulses do not occur in response to subtle perturbations of culture conditions.

**Fig 2:**
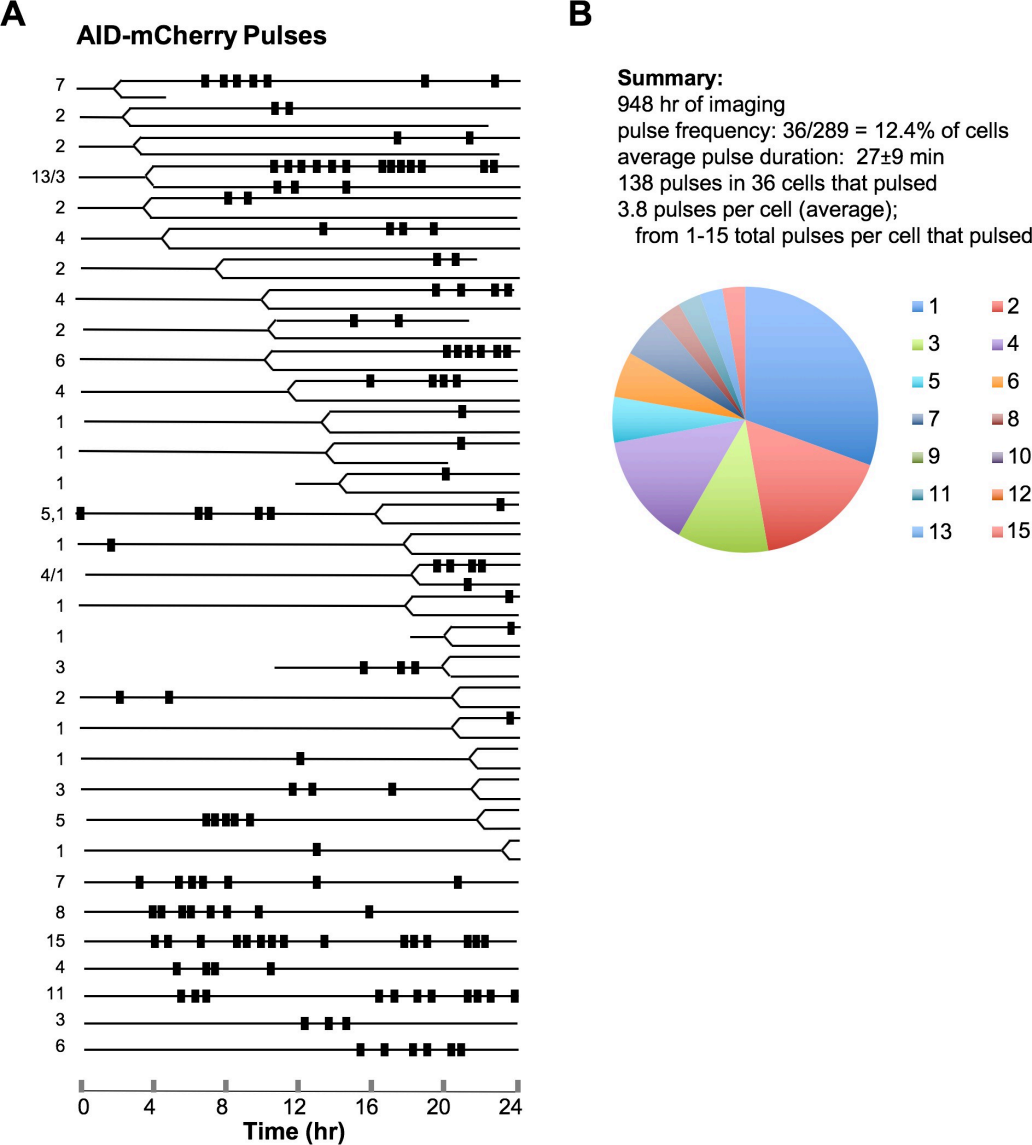
Timing and frequency of AID-mCherry nuclear pulses. (A) Timelines of nuclear pulses in HT1080 AID-mCherry transfectants relative to cell division. Diagrams are aligned to start of imaging (t = 0); and ranked by order of cell division (indicated by bifurcation of line). For each cell, AID-mCherry positive nuclear signals were scored and indicated as rectangles along the line denoting the 24 hr time course of live cell imaging. Lines are truncated prior to 24 hr for cells that exited the visual field. The number on the left indicates the number of pulses per cell. Commas (,) separate the tallies for mother and daughter cells and slashes (/) separate the tallies for daughter cells. (B) Above, summary of data on AID-mCherry pulses (see also S1 Table). Below, pie diagram indicating the number of cells with indicated number of pulses per cell (total number of cells = 36), based on the timeline in panel (A).

The timeline for pulsing in each cell was diagrammed to indicate when pulses occurred with respect to cell division, and individual timelines were ranked based on the interval between capture of the first image (t = 0) and cell division (Fig 2A). Most of the cells in which pulses were observed carried out cell division during the observation period (79%; S1 Table), evidence of their viability. Pulses occurred at variable intervals before and after cell division, so cell division does not regulate pulsing.

A total of 138 pulses were observed in the 36 cells that pulsed, or an average of 3.8 pulses in each cell that pulsed (S1 Table). Individual timelines contained from 1–15 AID-mCherry pulses (Fig 2B). Pulse duration averaged 27 ± 9 min (N = 138; S1 Table; S3A Fig). Duration did not change over the course of imaging and thus appeared to be independent of the number of pulses before or after a given pulse (S3A Fig). Intervals between pulses were irregular, although in some cells several pulses occurred in short succession, followed by an interval with no pulses. In 17 of the 26 timelines in which cell division was documented, both daughter cells could be followed during subsequent imaging. In most cases (15 of 17, or 88%), only one of the two daughter cells pulsed. In the two cases in which both daughter cells pulsed, pulses did not appear synchronized (Fig 2A). The timer that initiates the pulse thus appears not to be heritable.

### AID is not controlled by feedback regulation

Some proteins that pulse are under feedback regulation that maintains constant protein abundance despite changes in gene dosage or expression [32, 33, 39, 40]. If this is the case for AID, then the GFP and mCherry signals are predicted to be lower in HT1080 AID-mCherry AID-GFP double transfectants than in single transfectants. We established that the GFP and mCherry signals in double transfectants could be readily distinguished by flow cytometry (S3A Fig). We then showed that expression of AID-mCherry in AID-GFP transfectants did not diminish the GFP signal; and conversely, that expression of AID-GFP in AID-mCherry transfectants did not diminish the mCherry signal (S4B Fig). Thus, abundance of AID is not subject to feedback regulation.

### Pulses are independent of AID catalytic activity

**Fig 3:**
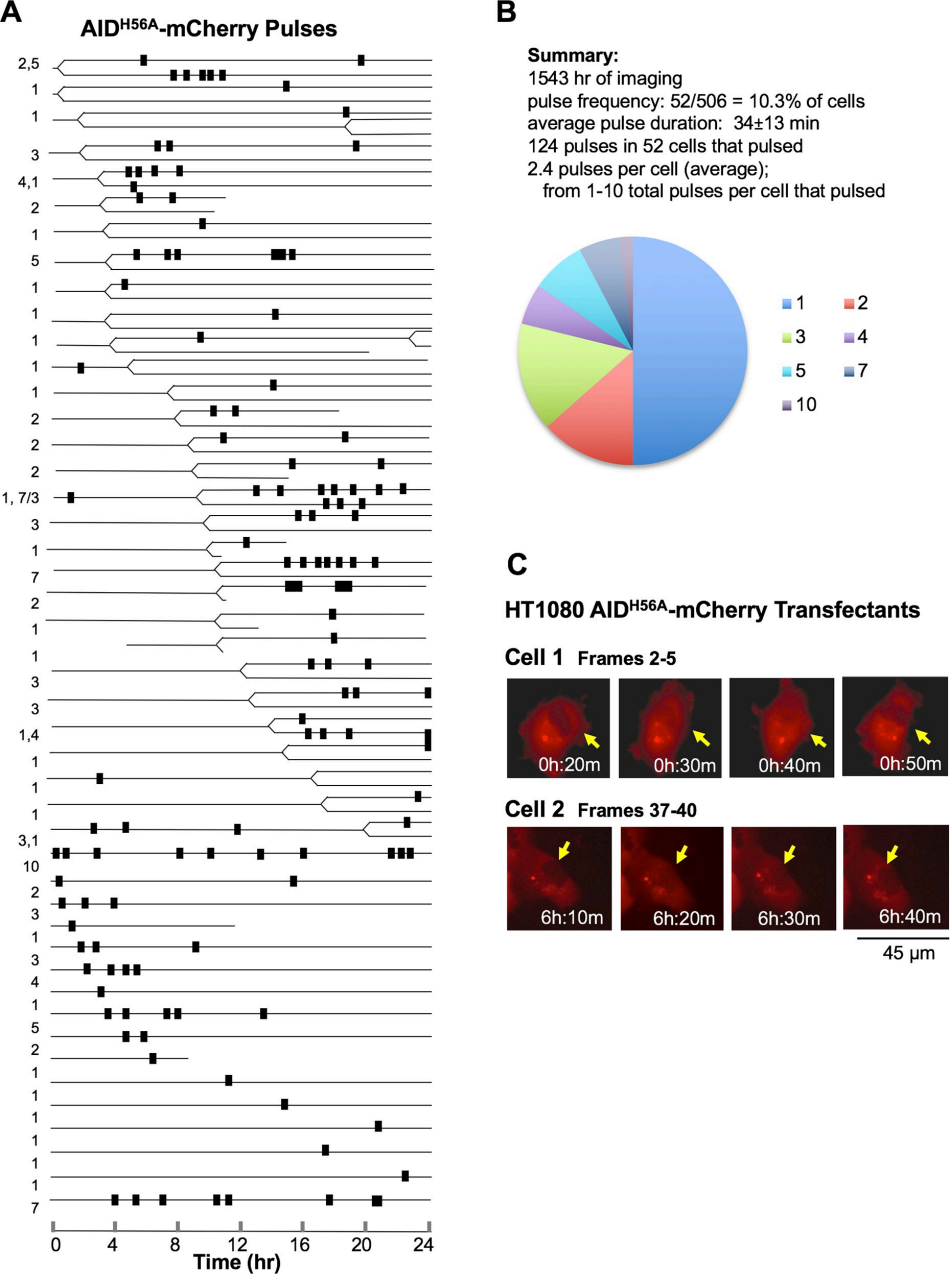
Nuclear pulses are independent of AID catalytic activity. (A) Timelines of nuclear pulses in HT1080 AID-mCherry transfectants relative to cell division. Details and notations as in Fig 2A. (B) Above, summary of data on AID^H56A^-mCherry pulses (see also S1 Table). Below, pie diagram indicating the number of cells with indicated number of pulses per cell (total number of cells = 46), based on the timeline in panel A. (C) Representative frames captured by live cell imaging of nuclear pulses in HT1080 AID^H56A^-mCherry transfectants at 10 min intervals. Arrows point to cells that pulse. Movies including these frames are provided in Supporting Information. Pulses captured in images above occur in: S6 Movie, cell at top center, frames 2–5 (upper images); S7 Movie, cell at center left of frames 37–40 (lower images).

AID localizes to the nucleus in response to DNA damage induced by ionizing radiation and other agents [28, 41, 42]. This raised the possibility that AID nuclear pulses are a response to damage caused by DNA deamination catalyzed by nuclear AID present at levels too low to detect by imaging. To test this, we asked if AID catalytic activity was necessary for nuclear pulses by analysis of HT1080 transfectants expressing AID^H56A^-mCherry. This derivative bears a mutation at a conserved histidine that coordinates an active site zinc ion essential for deaminase activity. This site has previously been targeted to impair deaminase activity because mutation of His to Ala at the corresponding residue of the active site of the highly related murine adenosine deaminase reduces catalytic activity without altering secondary or tertiary structure [27,43]. Live cell imaging of AID^H56A^-mCherry transfectants identified clear nuclear pulses (Fig 3; S6 and S7 Movies). Thus, nuclear pulses are independent of damage initiated by AID deaminase activity.

By time-lapse imaging of 506 individual HT1080 AID^H56A^-mCherry transfectants proliferating in asynchronous culture, at 10 min intervals over the course of 24 hr, we documented one or more nuclear pulses in 52 cells (S1 Table; S6 and S7 Movies). Pulses were not synchronous among the cells in a population. Timelines (46 total) of cell division and pulsing were generated in a total of 1543 hr of imaging (Fig 3A). Most of the cells analyzed (30/46, 65%) divided during the observation period (S1 Table). Individual cells exhibited from 1–10 pulses, with a significant fraction of the cells (26/52, 50%) pulsing only once (Fig 3B). Pulse duration averaged 34 ± 13 min (N = 124; S1 Table; S3B Fig). The average number of pulses (among cells that pulsed) was 2.4 per cell (124 pulses/52 cells), significantly fewer than the 3.8 pulses per cell in AID-mCherry transfectants (p = 0.03). We previously showed that mutation of AID catalytic activity (H56A or H56R) reduced both nuclear and cytoplasmic levels of AID to about 25% those of wild type AID [27], as is evident in representative images of individual examples of AID^H56A^-mCherry nuclear pulses (Fig 3C). That fewer pulses per cell were documented in HT1080 AID^H56A^-mCherry transfectants could reflect difficulty in scoring pulses with a fainter nuclear signal. Alternatively, AID catalytic activity may be a positive regulator of pulse frequency, even though it is not required for pulsing.

### AID pulses are coordinated with attenuation of signal of AID^F193A^, deficient in CRM1-mediated nuclear export

Pulses limit AID access to the nucleus and this will limit DNA deamination, providing a physiologic check on DNA damage potentially caused by AID. High content screening (HCS) microscopy has identified roles for both nuclear export and proteolysis in regulating AID nuclear levels in Ramos human B cells [27]. We asked if both mechanisms are also active in HT1080 cells by using HCS microscopy to analyze nuclear and cytoplasmic signals in populations of cells treated with MG132, which inhibits ubiquitin-dependent proteolysis; LMB, which inhibits CRM1-mediated nuclear export; or both (S8 and S9 Movies). Treatment of HT1080 AID-mCherry transfectants with MG132 alone had little effect. Treatment with LMB alone caused nuclear accumulation of AID-mCherry in all cells. Treatment with both LMB+MG132 caused a much greater increase in nuclear abundance than treatment with LMB alone, but did not affect cytoplasmic abundance. In HT1080 cells, the rate of AID nuclear degradation was comparable in all phases of cell cycle (S5C and S5D Fig). This contrasts with Ramos B cells, where nuclear proteolysis of AID is slightly slower in G1 phase than in S-G2/M phase [27]. Thus, both nuclear export and nuclear proteolysis regulate AID in HT1080 cells, and either process could in principle purge nuclear AID to terminate a pulse.

To further examine the role of nuclear export in terminating AID pulses, we carried out live cell imaging of HT1080 transfectants expressing AID^F193A^-mCherry, which carries a mutation in the NES that prevents nuclear export via the CRM1-mediated pathway. In normally proliferating cells, AID^F193A^-mCherry was absent from the cytoplasm and produced a strong nuclear signal on fluorescence imaging [27, 44]. The mCherry signal in cell populations expressing AID^F193A^-mCherry was lower than in cell populations expressing AID-mCherry (S6A Fig). By time-lapse imaging at 10 min intervals over the course of 24 hr, we recorded 568 individual HT1080 AID^F193A^-mCherry transfectants (1384 hr total imaging time; S8 and S9 Movies). A strong nuclear mCherry signal was evident in most cells, and there were no transient increases in signal as observed in the pulses; instead, transient attenuation of the AID^F193A^-mCherry signal was evident, occurring one or more times in 49 of the single cells imaged (8.6%; S1 Table). The reduced nuclear levels of AID^F193A^-mCherry could reflect regulation by several different mechanisms, including enhanced cytoplasmic retention, impaired nuclear entry, nuclear exit by passive diffusion or a CRM1-independent active pathway, or enhanced nuclear proteolysis.

**Fig 4:**
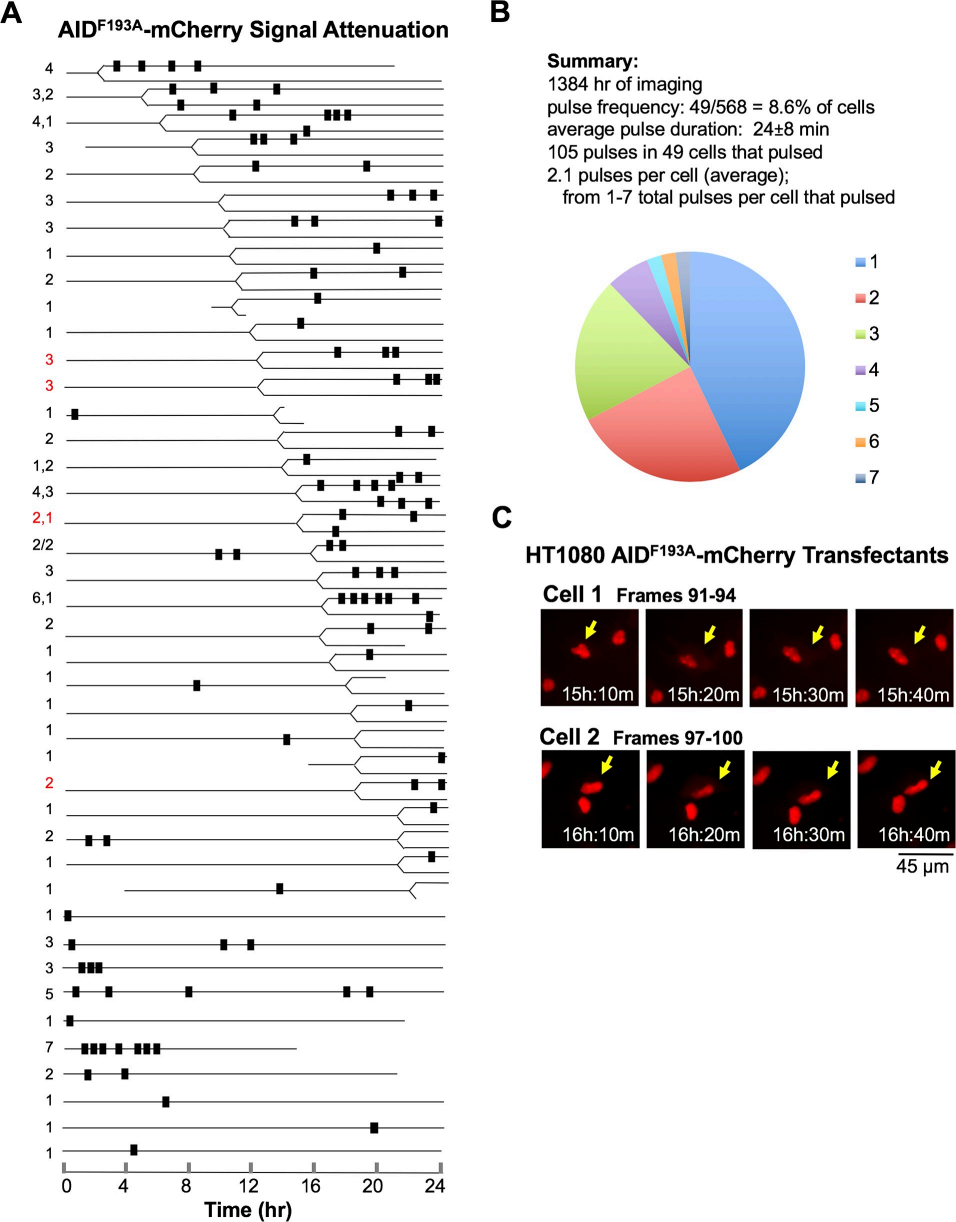
Timing and frequency of attenuation of the AID^F193A^-mCherry nuclear signal. (A) Timelines of attenuation events that reduce the nuclear signal in HT1080 AID^F193A^-mCherry transfectants. Details and notations as in Fig 2A. (B) Above, summary of data on AID^F193A^-mCherry pulses (see also S1 Table). Below, pie diagram showing the number of cells with indicated number of pulses per cell (total number of cells = 49), based on the timeline in panel (A). (C) Frames captured by live cell imaging showing attenuation of AID^F193A^-mCherry nuclear signal. Arrows point to cells in which signal attenuation was observed. Attenuation events captured in images occur in: S8 Movie, cell at upper left of frames 91–94 (left images); S9 Movie, cell at top center of frames 97–100 (right image; this cell undergoes division at frame 88).

A total of 105 signal attenuation events were documented. Timeline analysis of individual cells (Fig 4A) showed that most cells in which signal attenuation occurred also divided during the course of observation (78%; S1 Table). Attenuation events occurred at variable intervals after cell division, so division does not set the timer for signal attenuation. Individual cells exhibited 1–7 attenuation events (Fig 4B), an average of 2.1 per cell (105 events/49 cells). The average duration of AID^F193A^-mCherry signal attenuation events was 24 ± 8 min (N = 42; S1 Table; S3C Fig). Individual examples of transient attenuation of the AID^F193A^-mCherry nuclear signal are shown in representative images (Fig 4C).

### AID nuclear pulses and AID^F193A^ nuclear attenuation occur in synchrony

**Fig 5:**
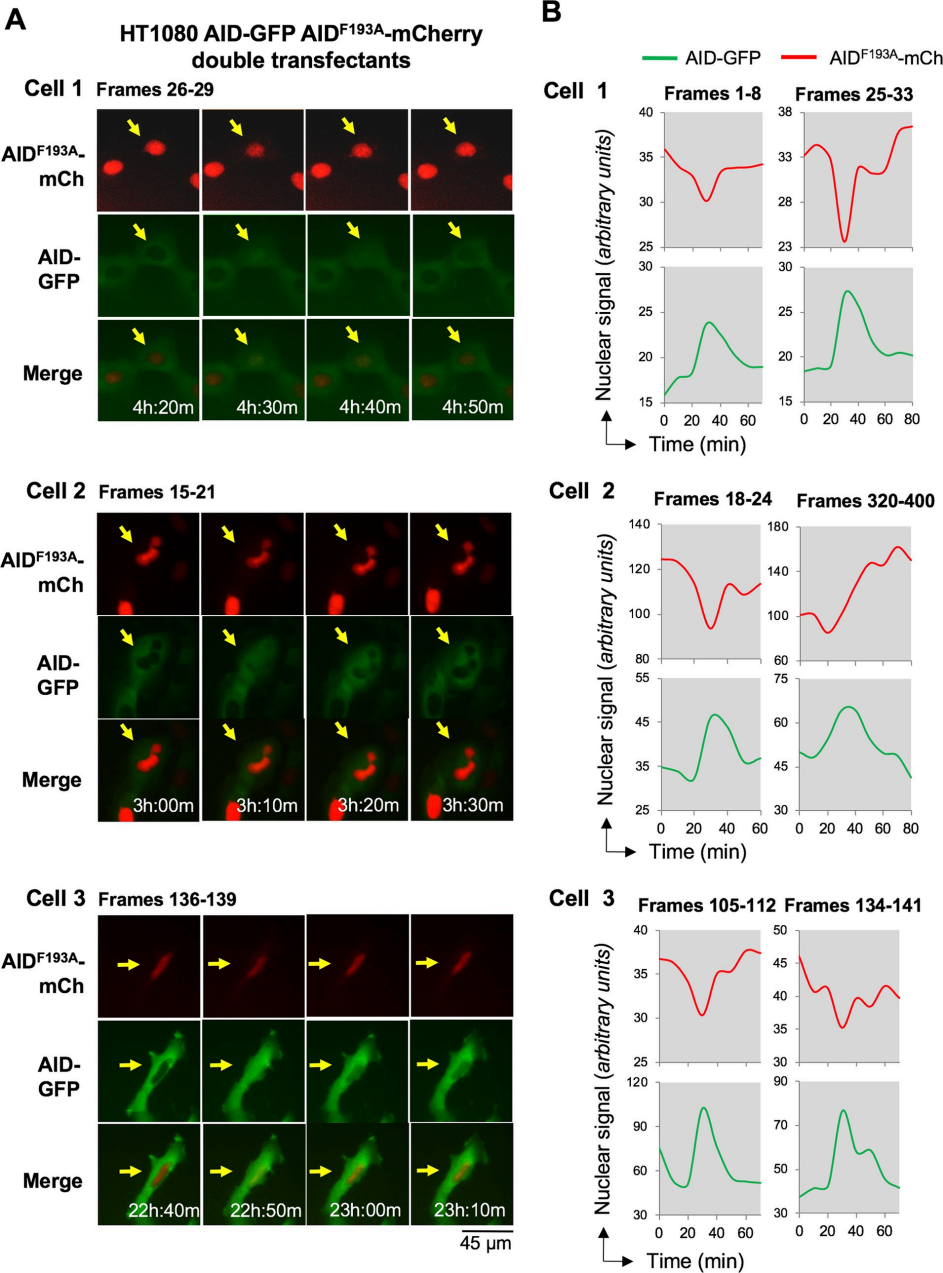
Attenuation of the AID^F193A^-mCherry nuclear signal occurs in synchrony with AID-GFP pulses. (A) Frames captured by live cell imaging, showing AID-GFP (top), AID^F193A^-mCherry (middle), and merged (bottom) images over the course of a pulse. Arrows point to cell that pulses. Pulses captured in images occur in: Cell1, S10 Movie, cell at top left of frames 26–29; Cell 2, S10 Movie, cell at center of frames 17–20; Cell 3, S11 Movie, cell at top center of frames 136–139, which undergoes division in frame 100. (B) Tracings of nuclear signals during two separate pulses spanning indicated frames for each of the three cells shown in panel (A).

The duration and frequency of AID-mCherry nuclear pulses and AID^F193A^-mCherry nuclear attenuation events are similar, suggesting that they may be regulated by shared mechanisms. If so, then pulses and attenuation events are predicted to occur in synchrony. To test this, we imaged HT1080 AID-GFP AID^F193A^-mCherry double transfectants, after first confirming by flow cytometry that the signals from these two fluorescent proteins could be readily distinguished, and that there was no change in mCherry signal in AID^F193A^-mCherry + AID-GFP double transfectants relative to AID^F193A^-mCherry transfectants; nor any effect on GFP signal in AID^F193A^-mCherry AID-GFP double transfectants relative to AID-GFP transfectants (S6B Fig). Time lapse imaging of live cells showed that AID-GFP nuclear pulses coincided with attenuation of the AID^F193A^-mCherry nuclear signal, as evident in representative examples from three different cells (Fig 5A; corresponding movies: S10 and S11 Movies).

To quantify the changes in the AID-GFP and AID^F193A^-mCherry nuclear signals, we determined the average mCherry and GFP signals in the nucleus and the cytoplasm (pixel intensities/area) in HT1080 AID-GFP and AID^F193A^-mCherry double transfectants. The GFP and mCherry signals were used to define the cell perimeter and nuclear boundary, respectively. Analysis of a total of 15 HT1080 AID-GFP AID^F193A^-mCherry double transfectants showed that of the 31 nuclear pulses/attenuation events that were observed, 94% were in synchrony. This synchrony is illustrated by representative tracings that quantify the nuclear signals of AID-GFP and AID^F193A^-mCherry for two pulses in each of the three cells shown in Fig 5A. The tracings reveal that over the course of a pulse, the AID-GFP nuclear signal increased 25% or more, then dropped; while the AID^F193A^-mCherry signal dropped by 20% or more, then recovered.

Timing of AID-GFP pulses and AID^F193A^-mCherry attenuation events appears to be coordinated. The maximum AID-GFP signal coincided with the minimum AID^F193A^-mCherry (Fig 5B). Similarly, the peak in the ratio of nuclear to cytoplasmic signal (N/C) for AID-GFP coincided with the minimum for AID^F193A^-mCherry (S7 Fig, above).

Tracings of cytoplasmic signals revealed a typically modest (≈20%) increase in the cytoplasmic signal of AID-GFP and AID^F193A^-mCherry during each pulse or attenuation event (Fig 5B; S7 Fig, below). This increase could reflect cytoplasmic accumulation of protein that has exited the nucleus or cytoplasmic retention of newly synthesized protein. The AID^F193A^ mutant is deficient in CRM1-dependent nuclear export, so if exit from the nucleus is responsible for AID^F193A^-mCherry signal attenuation and cytoplasmic protein increase, exit would need to occur via a CRM1-independent transport pathway or by passive diffusion. Nonetheless, it is important to recognize that synchrony of AID pulses and AID^F193A^ extinction events does not necessarily imply a common mechanism.

### P53 accumulates in the nucleus in response to AID pulses

The evidence for temporal coordination of AID-GFP pulses and AID^F193A^-mCherry attenuation events raised the question of whether AID pulses might also coordinate with nuclear import of P53. P53 is a key regulator of apoptosis. P53 nuclear levels rise in response to DNA damage and other oncogenic stresses, such as stress caused by impaired nucleolar function, and elevated levels induce a transcriptional program that results in apoptosis and cell death. To ask if accumulation of P53 might be coordinated with AID pulses, we generated AID-mCherry P53-GFP double transfectants of the SV40-transformed normal human fibroblast line, GM639. Live cell imaging of GM639 AID-mCherry P53-GFP double transfectants showed that nuclear P53-GFP levels increased in coordination with the initiation of the AID-mCherry pulse (S12 and S13 Movies). We analyzed 63 AID-mCherry P53-GFP double transfectants and identified AID nuclear pulses in 8 of them (12.6%). All of the cells in which AID-mCherry pulses occurred also exhibited an increase in nuclear P53-GFP signal. The nuclear P53-GFP signal was constant in 74.5% of cells that did not pulse in the course of imaging (41 of 55) and increased in 25.5% (14 out of 55).

**Fig 6:**
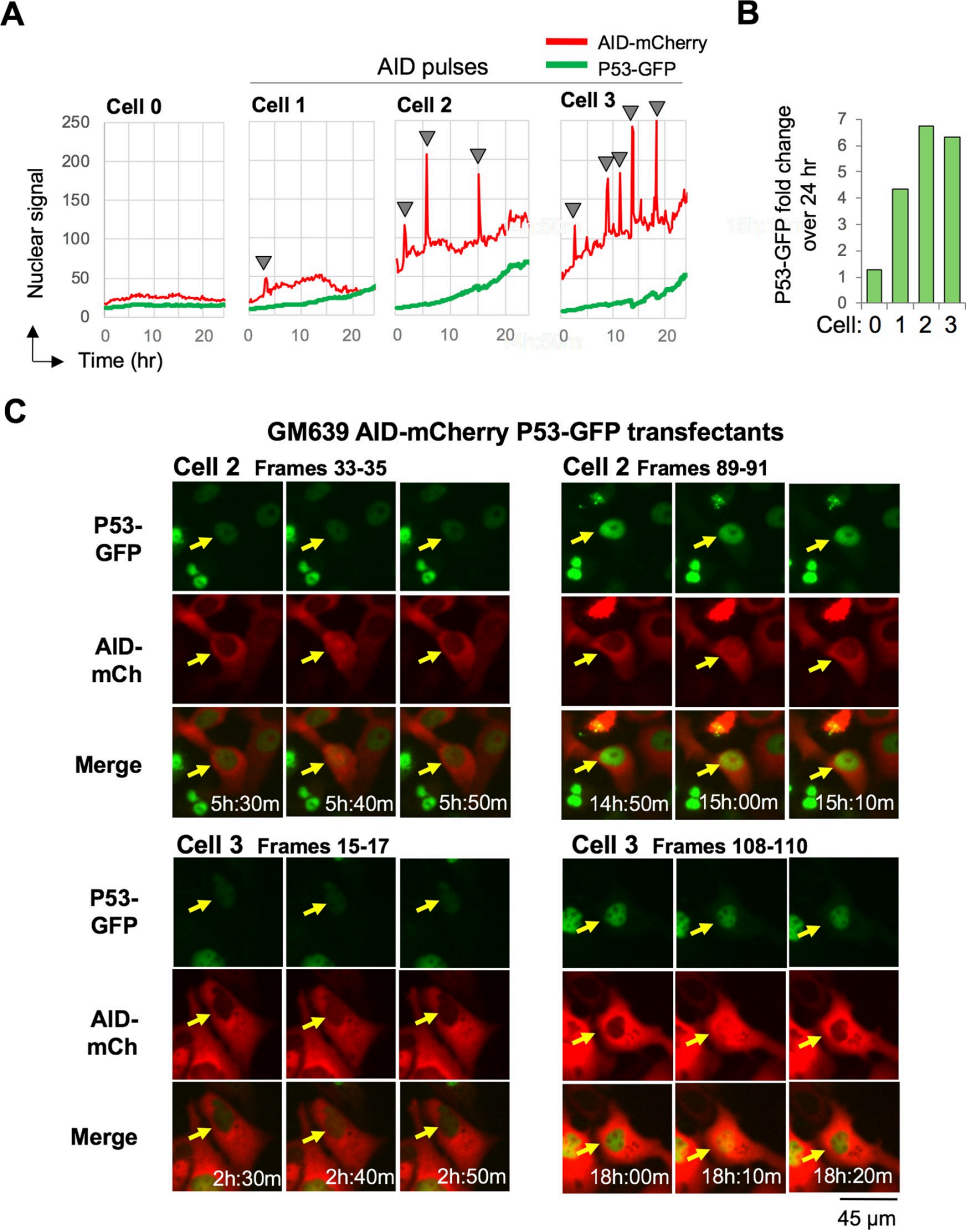
Nuclear P53 increases in response to nuclear pulses of AID. (A) Tracings of nuclear fluorescent signals of four GM639 AID-mCherry P53-GFP double transfectants over the duration of 24 hr time lapse imaging. Cell 0 did not pulse; cells 1–3 exhibited one, three and five pulses, respectively, indicated by arrowheads. (B) Quantification of fold change in nuclear P53-GFP signal in Cells 0, 1, 2 and 3 over the course of 24 hr of imaging. (C) Frames captured by live cell imaging, showing P53-GFP (top), AID-mCherry (middle), and merged (bottom) images of Cells 2 and 3 over the course of two pulses, the second and third pulse in Cell 2 and the first and fifth pulse in Cell 3. Arrows point to the cell that pulses. Pulses occur in: Cell 1, S12 Movie, cell at bottom center of frames 18–22; Cell 2, S13 Movie, cell at top right of frames 33–35 for the second pulse and frames 89–91 for the third pulse; and Cell 3, S13 Movie, cell at top right of frames 15–17 for the first pulse and 108–110 for the fifth pulse.

Fig 6A diagrams AID-mCherry and P53-GFP signals over the course of 24 hr imaging in one representative cell that did not pulse (Cell 0) and three cells that exhibited one, three or five AID-mCherry pulses as quantified by Image J analysis (Cells 1–3, respectively). Nuclear P53-GFP levels increased 4- to 6-fold in cells that pulsed (Fig 6B). Representative time-lapse images document temporal coordination between AID-mCherry pulses and P53-GFP accumulation (Fig 6C). Nuclear accumulation of P53-GFP is therefore temporally coordinated with AID nuclear pulses.

## Discussion

By imaging single living cells, we have shown that AID appears in the nucleus in brief (30 min) pulses in about 10% of cells. Pulses are a general rather than cell type-specific event, as they were observed in human HT1080 fibrosarcoma cells, GM639 transformed fibroblasts, and Ramos B cells. AID pulses do not depend upon AID catalytic activity, as the AID^H56A^ catalytic mutant pulsed with a frequency and duration comparable to wild type AID. Pulses occurred asynchronously among populations of proliferating cells, so pulses do not reflect a response to subtle changes in culture conditions, and they appear not to be cell cycle regulated.

Nuclear pulses are well-documented regulatory process, with the potential to coordinate nuclear entry of factors with complementary functions and to limit nuclear access of potent factors that regulate sensitive processes [32–34]. Pulsing controls nuclear access and persistence of about a dozen different transcriptional regulators in yeast and five from human cells, including P53 [35], NF-κB [36–38], ERK [45], Smad [46], and NFAT [47], all of which are key regulators that respond to and integrate inputs from multiple other factors and networks. AID fits naturally into this constellation of proteins, making it unlikely that AID pulses are an artefact of overexpression or tagged protein. The other proteins this far shown to pulse do so in response to specific stimuli or treatments: P53 pulses in response to DNA damage caused by ionizing radiation or drugs [35]; NF-κB in response to TNF [36–38]; ERK in response to EGF [45]; Smad in response to TGF-β [46]; and NFAT in response to changes in calcium concentration [47]. Some proteins that pulse do so in response to feedback regulation that maintains constant protein nuclear abundance [32, 33, 39, 40], but feedback regulation appears not to regulate AID pulses.

Analysis of 1363 individual cells over 3875 hours of imaging identified 367 pulses or attenuation events in 137 cells expressing wild-type AID and its AID^H56A^ and AID^F193A^ mutant derivatives, defective in catalysis and CRM1-dependent nuclear export, respectively (summarized in S1 Table). The average duration of events ranged from 24–34 minutes, and the frequency of cells in which events were observed ranged from 9–12%. The temporal coordination of nuclear pulses and attenuation events in double AID-GFP AID^F193A^-mCherry transfectants suggests that there is a common underlying cause for both pulses and attenuation events, but does not necessarily mean that the mechanism is shared. Altered regulation of cytoplasmic retention, nuclear import, nuclear export and proteolysis could all contribute to characteristic cycle of pulses and attenuation events. The results reported here do not enable us to distinguish among these mechanisms.

AID nuclear pulses are consistent with the physiological challenges of carrying out limited and highly regulated mutagenesis, although the results presented here do not identify a direct mechanistic connection between AID pulsing and function. The short duration of AID pulses will limit the interval during which DNA deamination can occur and thereby limit the potential for AID to cause DNA damage that exceeds its physiological mission. Intervals between pulses may provide time for DNA repair, or for assessment of whether sufficient deamination has occurred to support robust gene diversification. The relatively infrequent occurrence of AID pulses could also explain the paradoxical predominately cytoplasmic localization of AID evident in a variety of cells and tissues [14–18]. In addition, the episodic nature of AID pulses may influence repair pathways available to AID-induced damage. This should be taken into account in engineering applications that use AID as a mutagen, or when AID-induced damage is used as a model for repair at DNA nicks or strand breaks.

P53 accumulation in the nucleus accompanied AID pulses. This suggests that P53 may respond to DNA damage caused during a pulse, but P53 also enters the nucleus in response to other signals, including metabolic and oxidative stress and altered ribosome biogenesis. P53 accumulated in the nucleus of 25.5% of cells independent of an AID pulse, raising the possibility that accumulation may have occurred as a result of some other stress signal, perhaps even as a side-effect of imaging. Better understanding of regulation of AID pulses will clarify the role of DNA damage in initiating AID pulses.

AID is both a mutagen and a transcriptional regulator, as its deaminase activity can be deployed not only to initiate gene diversification, but also to erase epigenetic marks to alter gene expression patterns [9–13]. The other proteins that have been shown to pulse, in human cells and in yeast, all bind specific targets in duplex DNA to regulate transcription [32, 33, 39, 40]. The mechanisms that target AID to specific genes are not clearly defined. AID deaminates single-stranded but not double-stranded DNA, making actively transcribed genes targets for AID [48–50]. Pulsing adds a temporal cue to combinatorial regulation of transcription [34], and coordination of AID pulses and transcriptional activation could enhance targeting of some genes, such as Ig genes; or protect other genes from AID attack.

## Methods

### Expression constructs

The pEGFP-N3 construct for expression of AID-GFP was a gift from Dr. Javier Di Noia (Department of Microbiology and Immunology, University of Montreal, Montreal, Quebec, Canada). We substituted mCherry for a region of GFP flanked by ApaI and BsrGI restriction sites in the pEGFP-N3 construct to generate an AID-mCherry expression construct, pAID-mCh.

pAID-mCh CSII and pAID-GFP CSII: We amplified AID-mCherry and AID-GFP from pAID-mCh and pAID-GFP, respectively, with primers PQL31, 5’-ATATCAATTGAGATCCCAAATGGACAGCC-3’ and PQL32, 5’-ATATTCTAGATTACTTGTACAGCTCGTCCATGC-3’, and inserted the fragments between EcoRI and XbaI sites in p-mAG-GEM CSII, thereby replacing AG-GEM with AID-mCherry or AID-GFP.

p AID^F193A^-mCh CSII and p AID^H56A^ -mCh CSII: F193A and H56A mutants were generated using QuikChange II XL Site-Directed Mutagenesis Kit (Agilent, Cat # 200521) with primer set, F193A FOR 5’-CTTACGAGACGCAGCTCGTACTTTGGGAC-3’ and F193A REV 5’-GTCCCAAAGTACGAGCTGCGTCTCGTAAG-3’; H56A FOR 5’- GAACGGCTGCGCCGTGGAATTGCTC -3’ and H56A REV 5’- GAGCAATTCCACGGCGCAGCCGTTC -3’.

The P53-GFP expression construct was purchased from Addgene (plasmid #12091).

### Lentivirus production

Lentiviral particles were produced using second-generation packaging plasmids in 293T cells. 293T cells were transfected with transfer vector, viral packaging vector (psPAX2), and viral envelope vector (pMD2G) at 4:2:1 ratio using Lipofectamine LTX (Life Technologies, Cat # 15338100) transfection as directed by manufacturer’s protocol. Viral particles were collected at 24 hr and 48 hr after transfection and passed through 0.22 μm membrane (Steriflip; EMD Millipore; Cat # SCGP00525). Virus particles were used without further concentration.

### Cell culture, transduction and transfection

The human fibrosarcoma line HT1080 and SV40-transformed fibroblast line GM639 (ATCC) were cultured in DMEM media supplemented with 10% FBS, 2 mM L-glutamine, and penicillin/ streptomycin. HT1080 and GM639 cells were transfected by 4-D nucleofector (Lonza, Cat# PBC2-00675) and Lipofectamine LTX reagent (ThermoFisher Scientific, Cat# 15338100), respectively, as directed by the manufacturer.

The human Burkitt lymphoma cell line, Ramos, was cultured in RPM 1640 media supplemented with 10% FBS, 2 mM L-glutamine, penicillin/ streptomycin, 1X non-essential amino acids (Gibco, Cat# 11140–050), 1 mM sodium pyruvate (Gibco, Cat# 11360–070), and 10 mM HEPES (Gibco 15630–080). Lentiviral transductions used 2x10^5^ Ramos B cells grown in medium containing 8 μg/ml of polybrene per ml. Cells were grown for 3–4 days after transduction and then sorted for mCherry+ to enrich for transduced cells expressing the desired fluorescent protein prior, typically constituting 0.1–10% of the population.

Cells were treated with leptomycin B (LMB; LC Laboratories, Cat# L6100) at 50 ng/ml or MG132 (Z-Leu-Leu-Leu-aldehyde; Sigma-Aldrich) at 50 μM for indicated time.

High content screening microscopy and analysis of cell cycle were carried out as previously described [27].

### Live-cell imaging

Time-lapse microscopy was carried out using a DeltaVision microscope equipped with an environmental chamber to control for temperature (37°C) and CO_2_ (5% CO_2_). Transfected HT1080 and GM639 cells were grown in a 35 mm glass bottom dish (MatTek, sample part # P35G-0-10-C) at 37°C/5% CO_2_ at least 24 hr before imaging. HT1080 and GM639 cells were imaged at 10 min intervals for duration of 24 hr.

Ramos B cells expressing AID-mCherry were grown at 37°C/5% CO_2_ at least 24 hr before imaging on LiveCell Array Microscope slides (Nunc, Cat # 130505), which limited but did not prevent tumbling. B cells were imaged at 15 min intervals for 3–4 hr; more frequent or more extended imaging compromised cell survival.

Timelines illustrate pulses and attenuation events of less than 60 min duration relative to cell cycle. Longer events were occasionally observed in dying cells, and these were eliminated from the dataset. To indicate when events occurred with respect to cell division, individual timelines were ranked based on the interval between capture of the first image (t = 0) and separation of the daughter cells.

ImageJ software with “RGB measure” plugin was used to quantify nuclear and cytoplasmic signals of GFP and mCherry.

### High content screening (HCS) microscopy and analysis

HT1080 AID-mCherry transfectants were grown in 96-well μclear microplate (Greiner Bio One) for 24 hr prior to treatment with LMB, MG132, or both at indicated times. Following treatment, cells were fixed in 3.7% formaldehyde and stained with whole cell stain (HCS CellMask, Invitrogen) and DAPI. Fixed cells were then washed with PBS and were imaged by Thermo Scientific ArrayScan VTI HCS reader. Cells with very low or very high mCherry signals were eliminated, gating based on the mock transduction control (low) and eliminating cells with signals more than 5 SD from the mean (high). The HCS Colocalization BioApplication protocol was used to determine nuclear and whole cell boundaries in individual cells as defined by DAPI and HCS CellMask, respectively, thereby defining the cytoplasmic region as the region between nuclear and whole cell boundaries. The average signal in the nuclear and cytoplasmic compartments was determined in individual cells by measuring the total intensity of mCherry signal divided by area within each compartment. The ratio of nuclear to cytoplasmic signal (N/C) was calculated as the ratio of the average signals of nuclear and cytoplasmic mCherry.

### Statistical analysis

Statistical significance was determined using two-tailed, unpaired Student’s t-test, assuming unequal variances

## Supporting information

S1 Table Nuclear pulses in HT1080 AID-mCherry and HT1080 AID^F193A^-mCherry transfectants. Related to Figs 2, 3 and 4.
(TIF)

S1 Fig AID localizes throughout the cell during mitosis in HT1080 cells. Representative frames captured by live cell imaging, showing two HT1080 AID-mCherry transfectants at 10 min intervals. Arrows point to cells that undergo cell division and emerging daughter cells in still frames. Cell division captured in images shown occur in S1 Movie in a single cell at the center of frames 67–76 (upper images) and frames 80–89 (lower images).
(TIF)

S2 Fig AID pulses in the nucleus of Ramos B cells. Representative frames captured by live cell imaging, showing Ramos B cells AID-mCherry transductants at 15 min intervals. Arrows point to cells that pulse. Movies including these frames are provided in Supporting Information. Pulses captured in images occur in upper images, S4 Movie, frames 7–10, cell at center left; lower images, S5 Movie, frames 10–13, cell at center right. Note that these frames illustrate how the absence of stable attachments interferes with analysis of B cells by live cell imaging over extended time periods: during imaging, a cell moved into the lower left of the upper frames, and out of the upper left of the lower frames.
(TIF)

S3 Fig Duration of pulses in HT1080 AID-mCherry and AID^F193A^-mCherry transfectants. Average duration for each pulse, rank ordered from t = 0, the start of observation. Black bars represent SEM.
(A) HT1080 AID-mCherry transfectants.
(B) HT1080 AID^H56A^-mCherry transfectants.
(C) HT1080 AID^F193A^-mCherry transfectants.
(TIF)

S4 Fig Relative levels of AID-GFP and AID-mCherry in HT1080 transfectants, as determined by flow cytometry. (A) Scatter plots of PE-Texas Red (mCherry) and FITC (GFP) signals in HT1080 cells expressing indicated AID derivative(s). Mock, no transfection.
(B) Flow cytometry of indicated HT1080 transfectants, showing PE-Texas Red (mCherry) and FITC (GFP) signals relative to maximum.
(TIF)

S5 Fig Nuclear AID is sensitive to ubiquitin-dependent proteolysis in HT1080 cells. (A) Scatter plots of nuclear vs. cytoplasmic mCherry signals for HT1080 AID-mCherry transfectants, untreated (t = 0) or treated with MG132, LMB, or LMB+MG132 for 0.5, 1, 2 or 4 hr, as indicated.
(B) Quantification of nuclear and cytoplasmic AID-mCherry signal and N/C ratio, relative to untreated cells, at indicated times post-treatment with MG132, LMB, or both. Dotted line represents no change (fold change of 1). Each point represents a population average, and black bars (too small to be discerned readily) represent SEM of the population. Analysis was carried out by high content screening microscopy, as previously described [27].
(C) Representative analysis of kinetics of response of AID-mCherry nuclear (solid lines) and cytoplasmic (dashed lines) signals to treatment with MG132, LMB or LMB + MG132 in G1, S and G2/M phase cells. Each point represents a population average, and black bars represent SEM of the population, which are too small to discern. Dotted line represents no change (fold change of 1).
(D) Relative rates of nuclear degradation of AID-mCherry following LMB treatment in G1, S and G2/M phases. Rates were calculated as the slope of the line defined by the population averages at 1 and 2 hr of treatment. Values are relative to the slope in G1 phase.
(TIF)

S6 Fig Relative levels of AID-GFP, AID-mCherry, and AID^F193A^-mCherry signals in HT1080 transfectants, as determined by flow cytometry. (A). Scatter plots of mCherry and GFP signals in HT1080 cells expressing indicated AID derivative(s).
(B) Left, scatter plots of mCherry and GFP signals in HT1080 AID-GFP AID^F193A^-mCherry double transfectants. Right, flow cytometry of indicated HT1080 transfectants, showing mCherry and GFP signals relative to maximum.
(TIF)

S7 Fig Tracings of cytoplasmic signals and ratios of nuclear to cytoplasmic signals in HT1080 AID-GFP AID^F193A^-mCherry double transfectants. Above: Ratios of nuclear to cytoplasmic signals (N/C) for AID-GFP (green) and AID^F193A^-mCherry (red) in two pulses and synchronous attenuation events spanning indicated frames for each of the three cells shown in Fig 4. Control quantification of the AID-GFP and AID^F193A^-mCherry N/C ratio over a 60 min period when a cell was not pulsing yielded a relatively flat line, with frame-to-frame variations of <5% of total signal (far right). Arrows above tracings indicate times of peak N/C ratio for AID-GFP and of minimal N/C ratio for AID^F193A^-mCherry signal; which correspond to peak of AID^F193A^-mCherry cytoplasmic signal, above. Dotted line indicates nuclear/cytoplasmic signal ratio of one.
Below: Cytoplasmic signal tracings for intervals corresponding to tracings of nuclear signals spanning indicated frames for each of the three cells shown in Fig 4. Arrows in panels in top row indicate times of peak AID^F193A^-mCherry cytoplasmic signals.
(TIF)

S1 Movie Live cell imaging of HT1080 AID-mCherry transfectants. Movie is compressed into 29 seconds at total of 144 time lap frames, representing 24 hr (1440 min) of imaging. Each frame represents 10 min.
(MOV)

S2 Movie Live cell imaging of HT1080 AID-GFP mKO2-CDT1 double transfectants. Movie is compressed into 29 seconds at total of 144 time lap frames, representing 24 hr (1440 min) of imaging. Each frame represents 10 min.
(MOV)

S3 Movie Live cell imaging of HT1080 AID-GFP mKO2-CDT1 double transfectants. Movie is compressed into 29 seconds at total of 144 time lap frames, representing 24 hr (1440 min) of imaging. Each frame represents 10 min.
(MOV)

S4 Movie Live cell imaging of Ramos AID-mCherry transductants. Movie is compressed into 16 seconds a total of 16 time lapse frames, representing 4 hr (240 min) of imaging. Thus, each frame represents 15 min.
(MOV)

S5 Movie Live cell imaging of Ramos AID-mCherry transductants. Movie is compressed into 16 seconds a total of 16 time lapse frames, representing 4 hr (240 min) of imaging. Thus, each frame represents 15 min.
(MOV)

S6 Movie Live cell imaging of HT1080 AID^H56A^-mCherry transfectants. Movie is compressed into 29 seconds at total of 144 time lap frames, representing 24 hr (1440 min) of imaging. Each frame represents 10 min.
(MOV)

S7 Movie Live cell imaging of HT1080 AID^H56A^-mCherry transfectants. Movie is compressed into 29 seconds at total of 144 time lap frames, representing 24 hr (1440 min) of imaging. Each frame represents 10 min.
(MOV)

S8 Movie Live cell imaging of HT1080 AID^F193A^-mCherry transfectants. Movie is compressed into 29 seconds at total of 144 time lap frames, representing 24 hr (1440 min) of imaging. Each frame represents 10 min.
(MOV)

S9 Movie Live cell imaging of HT1080 AID^F193A^-mCherry transfectants. Movie is compressed into 29 seconds at total of 144 time lap frames, representing 24 hr (1440 min) of imaging. Each frame represents 10 min.
(MOV)

S10 Movie Live cell imaging of HT1080 AID-GFP AID^F193A^-mCherry double transfectants. Movie is compressed into 29 seconds at total of 144 time lap frames, representing 24 hr (1440 min) of imaging. Each frame represents 10 min.
(MOV)

S11 Movie Live cell imaging of HT1080 AID-GFP AID^F193A^-mCherry double transfectants. Movie is compressed into 29 seconds at total of 144 time lap frames, representing 24 hr (1440 min) of imaging. Each frame represents 10 min.
(MOV)

S12 Movie Live cell imaging of GM639 P53-GFP AID-mCherry double transfectants. Movie is compressed into 29 seconds at total of 144 time lap frames, representing 24 hr (1440 min) of imaging. Each frame represents 10 min.
(AVI)

S13 Movie Live cell imaging of GM639 P53-GFP AID-mCherry double transfectants. Movie is compressed into 29 seconds at total of 144 time lap frames, representing 24 hr (1440 min) of imaging. Each frame represents 10 min.
(AVI)
