# Correction: Activation-induced deaminase (AID) localizes to the nucleus in brief pulses

**DOI:** 10.1371/journal.pgen.1008310

**Published:** 2019-07-26

**Authors:** The PLOS Genetics Staff

## Notice of republication

This article was republished on March 26, 2019 to correct an error in the ORCID iD of the corresponding author introduced during the typesetting process. The publisher apologizes for the errors. Please download this article again to view the correct version.

## Supporting information

S1 File Originally published, uncorrected article. (PDF)

S2 File Republished, corrected article. (PDF)
