# Supplementary figures and images for: Activation-induced deaminase (AID) localizes to the nucleus in brief pulses
Source: PLoS Genet. 2019 Feb 27;15(2):e1007968. doi: 10.1371/journal.pgen.1007968 (PMC6411215; doi:10.1371/journal.pgen.1007968)

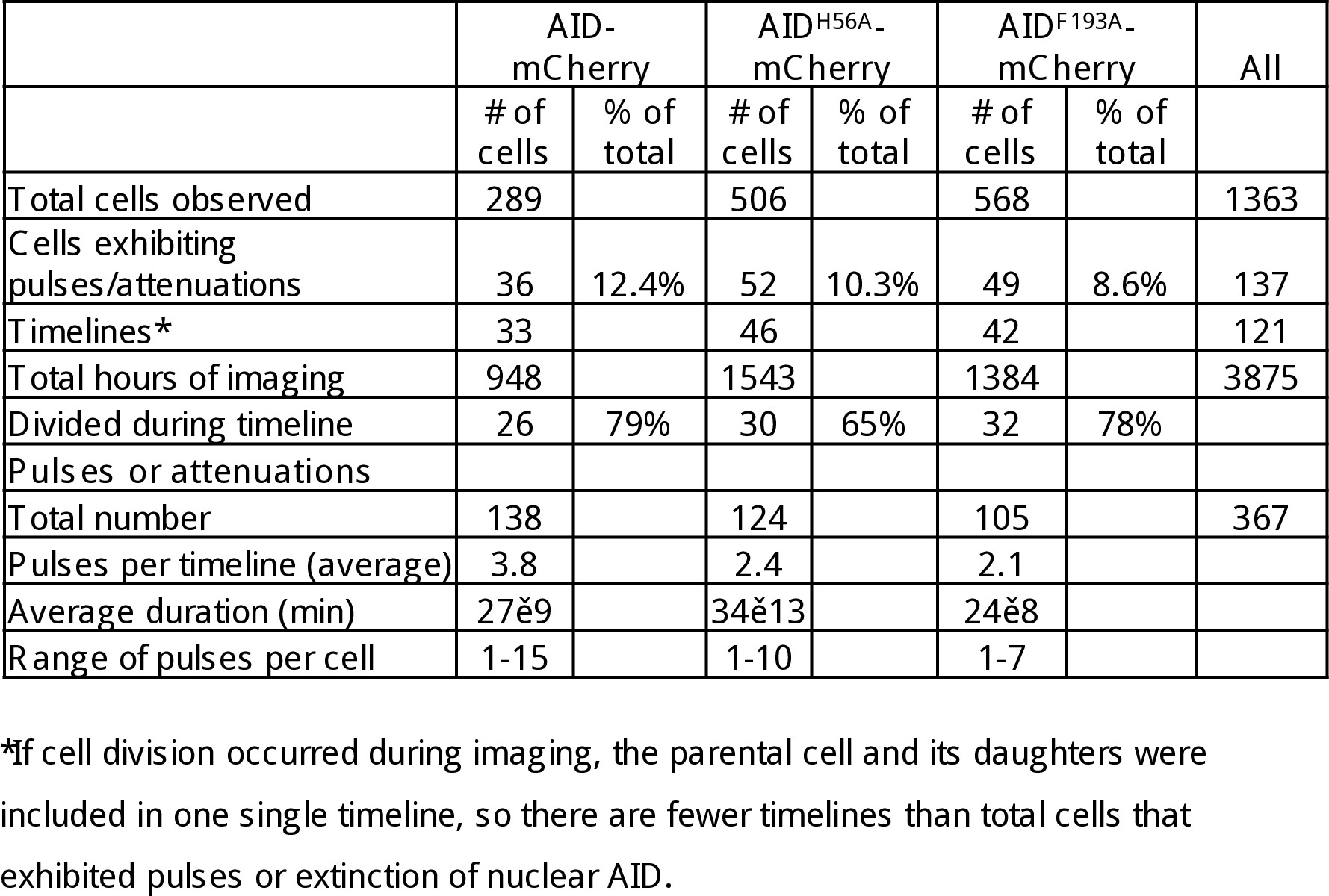

Supplement: S1 Table — Related to Figs 2, 3 and 4. (TIF) [file pgen.1007968.s001.tif]

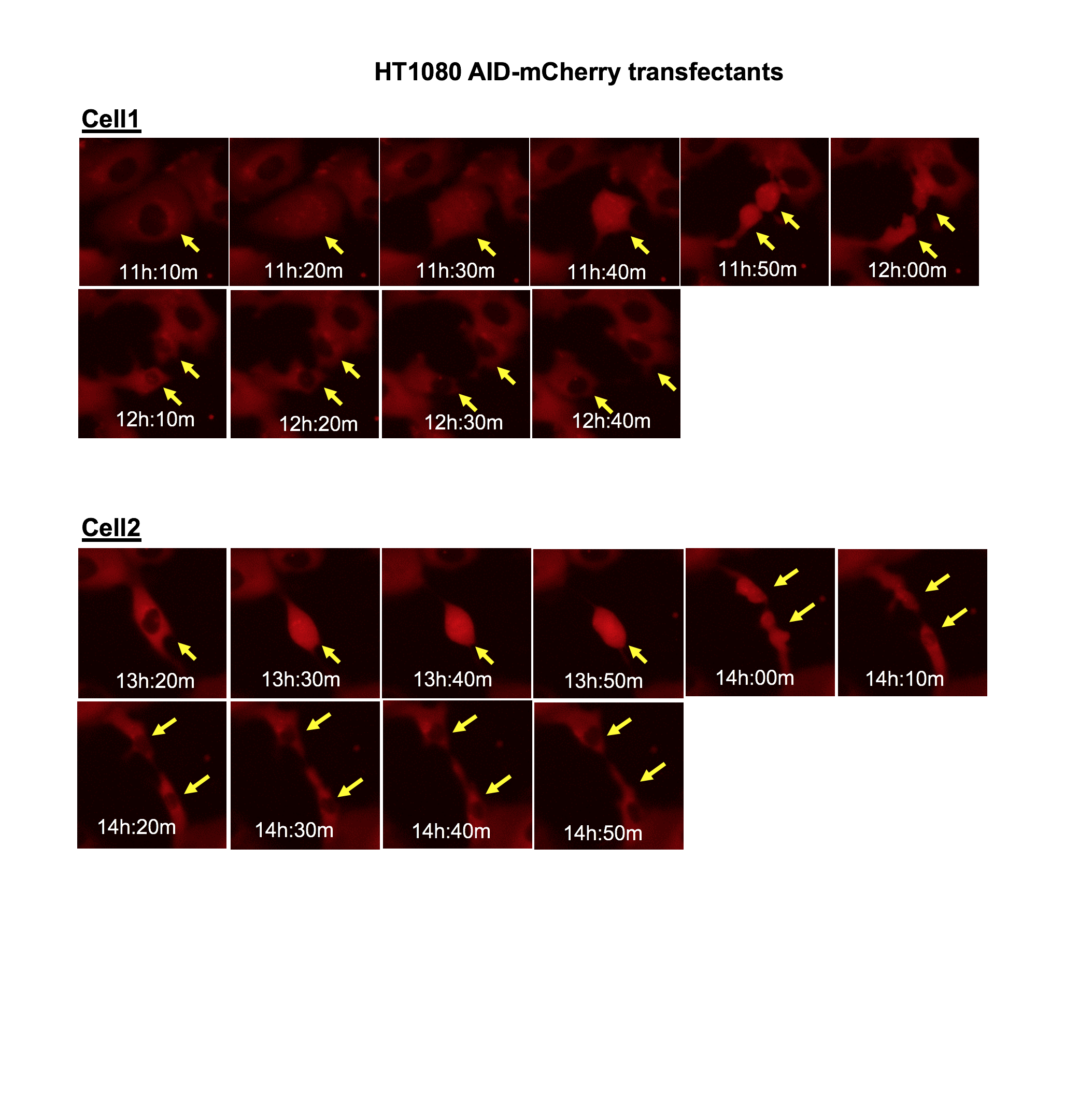

Supplement: S1 Fig — Representative frames captured by live cell imaging, showing two HT1080 AID-mCherry transfectants at 10 min intervals. Arrows point to cells that undergo cell division and emerging daughter cells in still frames. Cell division captured in images shown occur in S1 Movie in a single cell at the center of frames 67–76 (upper images) and frames 80–89 (lower images). (TIF) [file pgen.1007968.s002.tif]

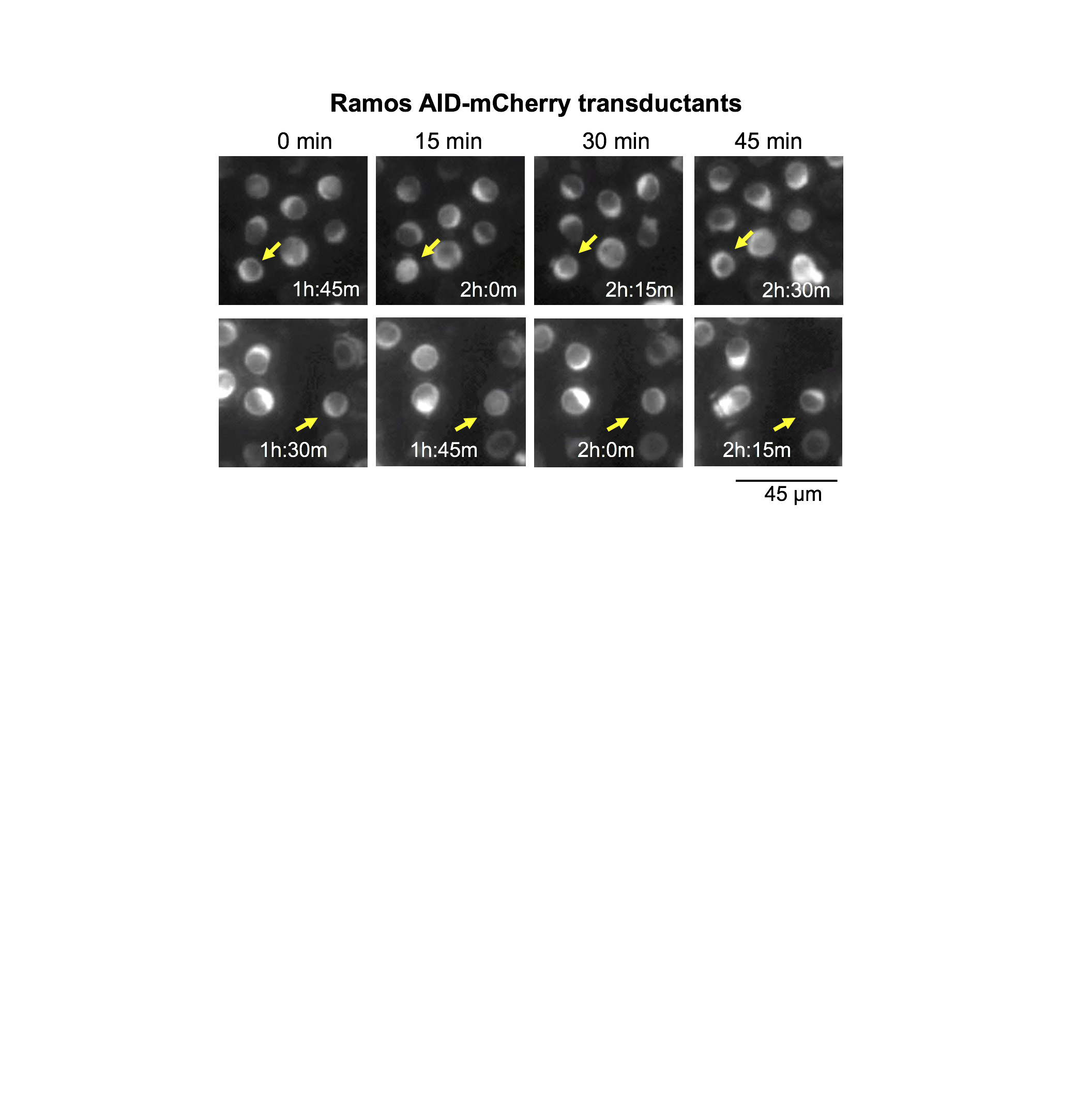

Supplement: S2 Fig — Representative frames captured by live cell imaging, showing Ramos B cells AID-mCherry transductants at 15 min intervals. Arrows point to cells that pulse. Movies including these frames are provided in Supporting Information. Pulses captured in images occur in upper images, S4 Movie, frames 7–10, cell at center left; lower images, S5 Movie, frames 10–13, cell at center right. Note that these frames illustrate how the absence of stable attachments interferes with analysis of B cells by live cell imaging over extended time periods: during imaging, a cell moved into the lower left of the upper frames, and out of the upper left of the lower frames. (TIF) [file pgen.1007968.s003.tif]

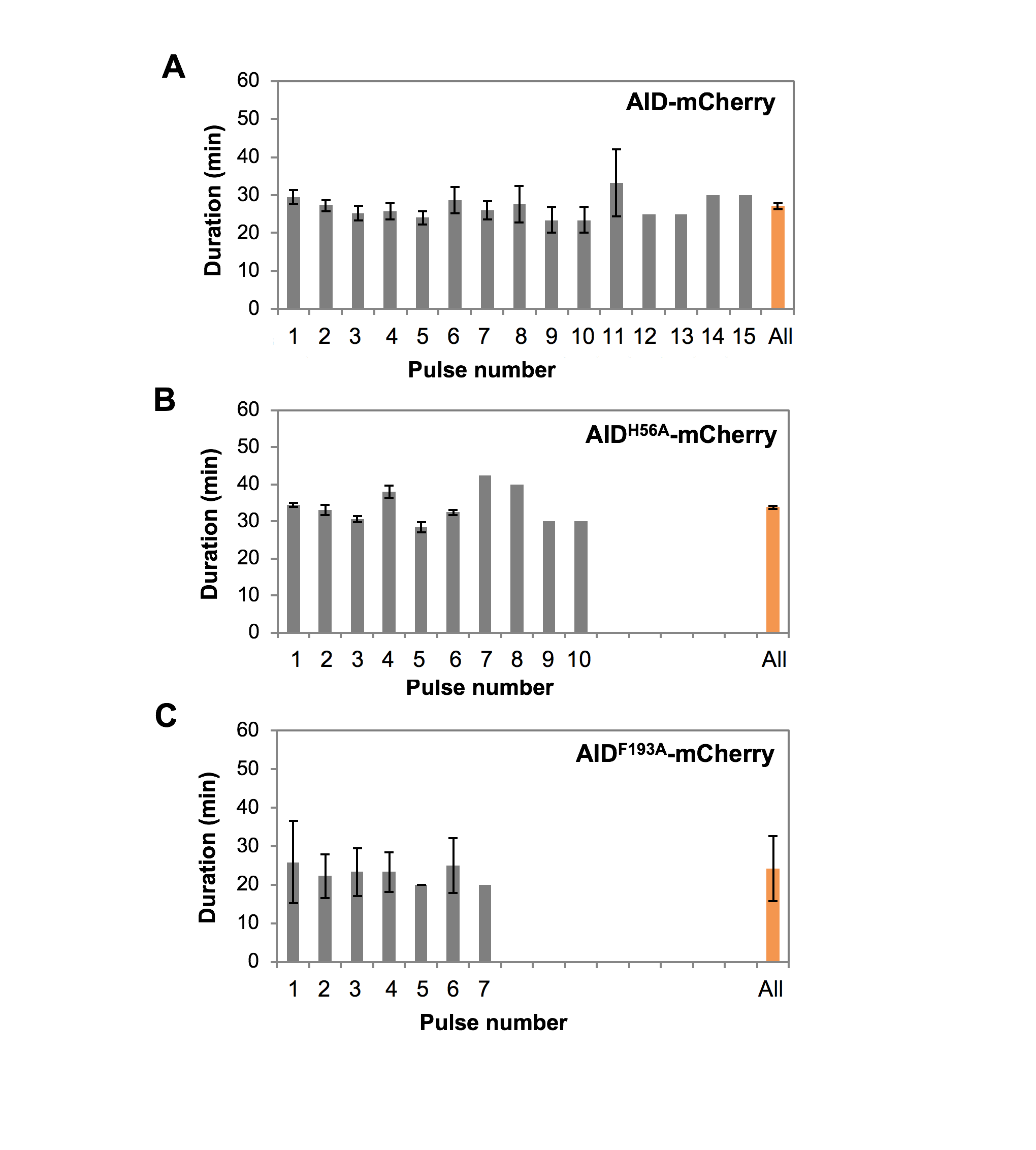

Supplement: S3 Fig — Average duration for each pulse, rank ordered from t = 0, the start of observation. Black bars represent SEM. (A) HT1080 AID-mCherry transfectants. (B) HT1080 AIDH56A-mCherry transfectants. (C) HT1080 AIDF193A-mCherry transfectants. (TIF) [file pgen.1007968.s004.tif]

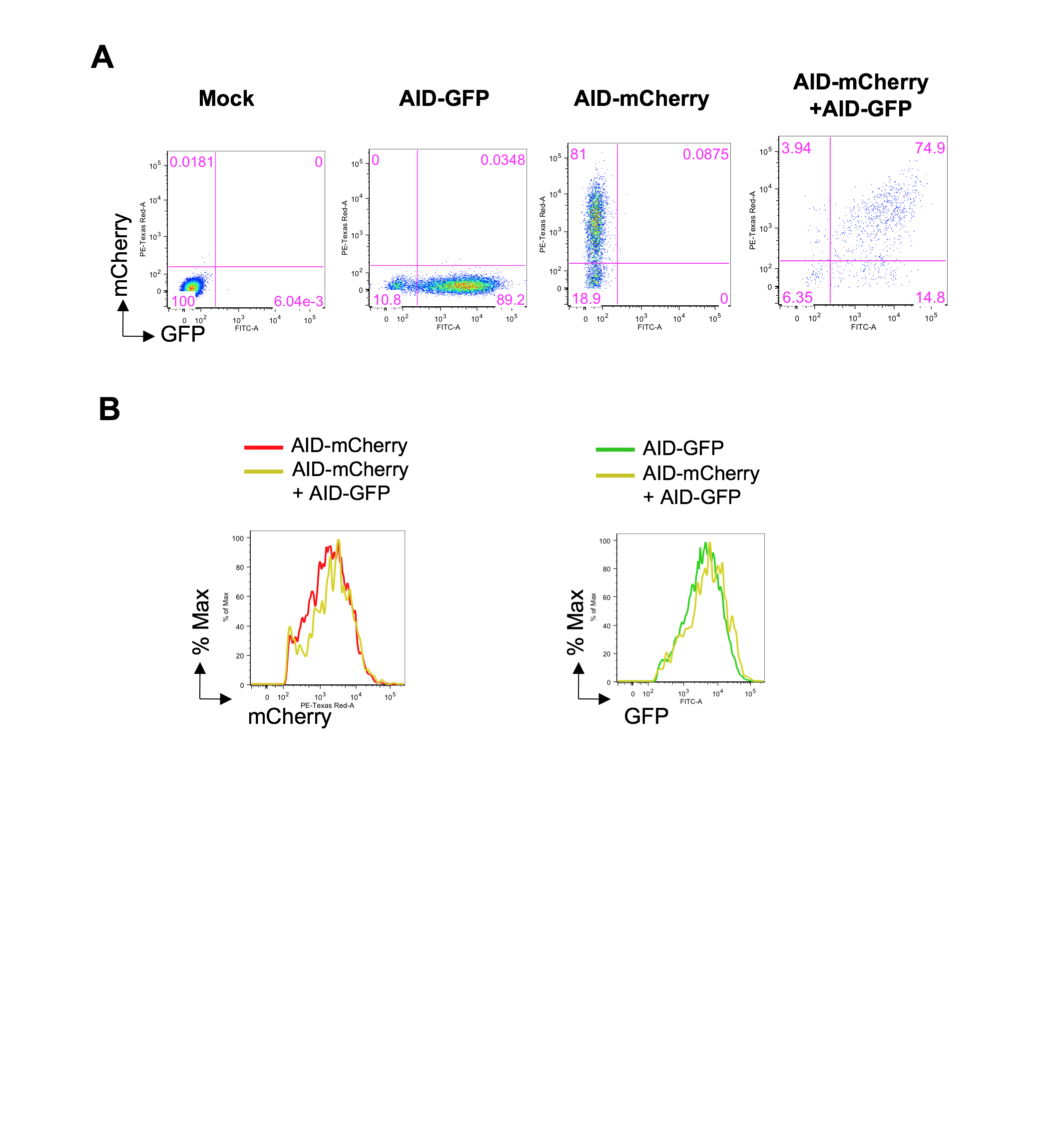

Supplement: S4 Fig — (A) Scatter plots of PE-Texas Red (mCherry) and FITC (GFP) signals in HT1080 cells expressing indicated AID derivative(s). Mock, no transfection. (B) Flow cytometry of indicated HT1080 transfectants, showing PE-Texas Red (mCherry) and FITC (GFP) signals relative to maximum. (TIF) [file pgen.1007968.s005.tif]

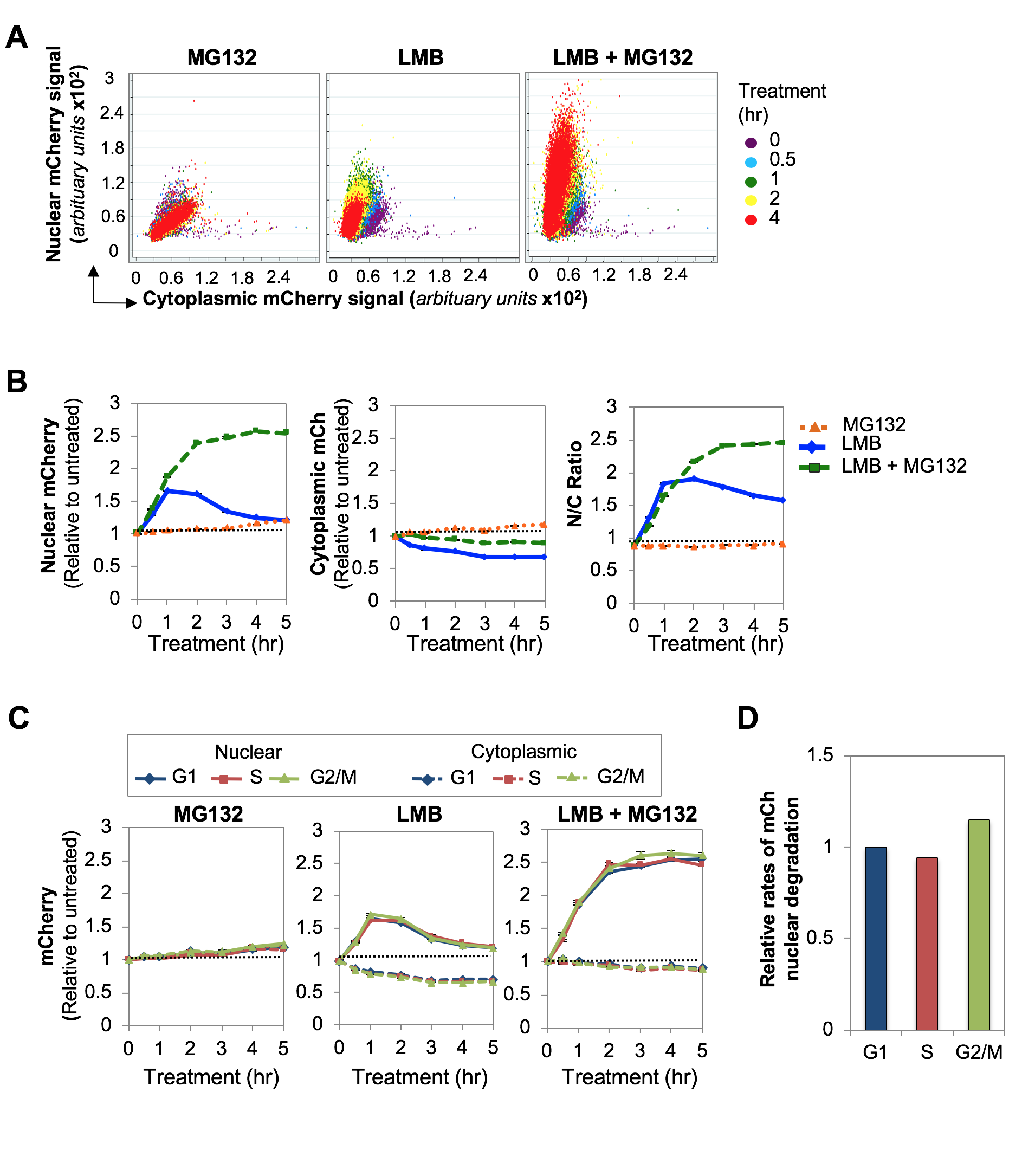

Supplement: S5 Fig — (A) Scatter plots of nuclear vs. cytoplasmic mCherry signals for HT1080 AID-mCherry transfectants, untreated (t = 0) or treated with MG132, LMB, or LMB+MG132 for 0.5, 1, 2 or 4 hr, as indicated. (B) Quantification of nuclear and cytoplasmic AID-mCherry signal and N/C ratio, relative to untreated cells, at indicated times post-treatment with MG132, LMB, or both. Dotted line represents no change (fold change of 1). Each point represents a population average, and black bars (too small to be discerned readily) represent SEM of the population. Analysis was carried out by high content screening microscopy, as previously described [27]. (C) Representative analysis of kinetics of response of AID-mCherry nuclear (solid lines) and cytoplasmic (dashed lines) signals to treatment with MG132, LMB or LMB + MG132 in G1, S and G2/M phase cells. Each point represents a population average, and black bars represent SEM of the population, which are too small to discern. Dotted line represents no change (fold change of 1). (D) Relative rates of nuclear degradation of AID-mCherry following LMB treatment in G1, S and G2/M phases. Rates were calculated as the slope of the line defined by the population averages at 1 and 2 hr of treatment. Values are relative to the slope in G1 phase. (TIF) [file pgen.1007968.s006.tif]

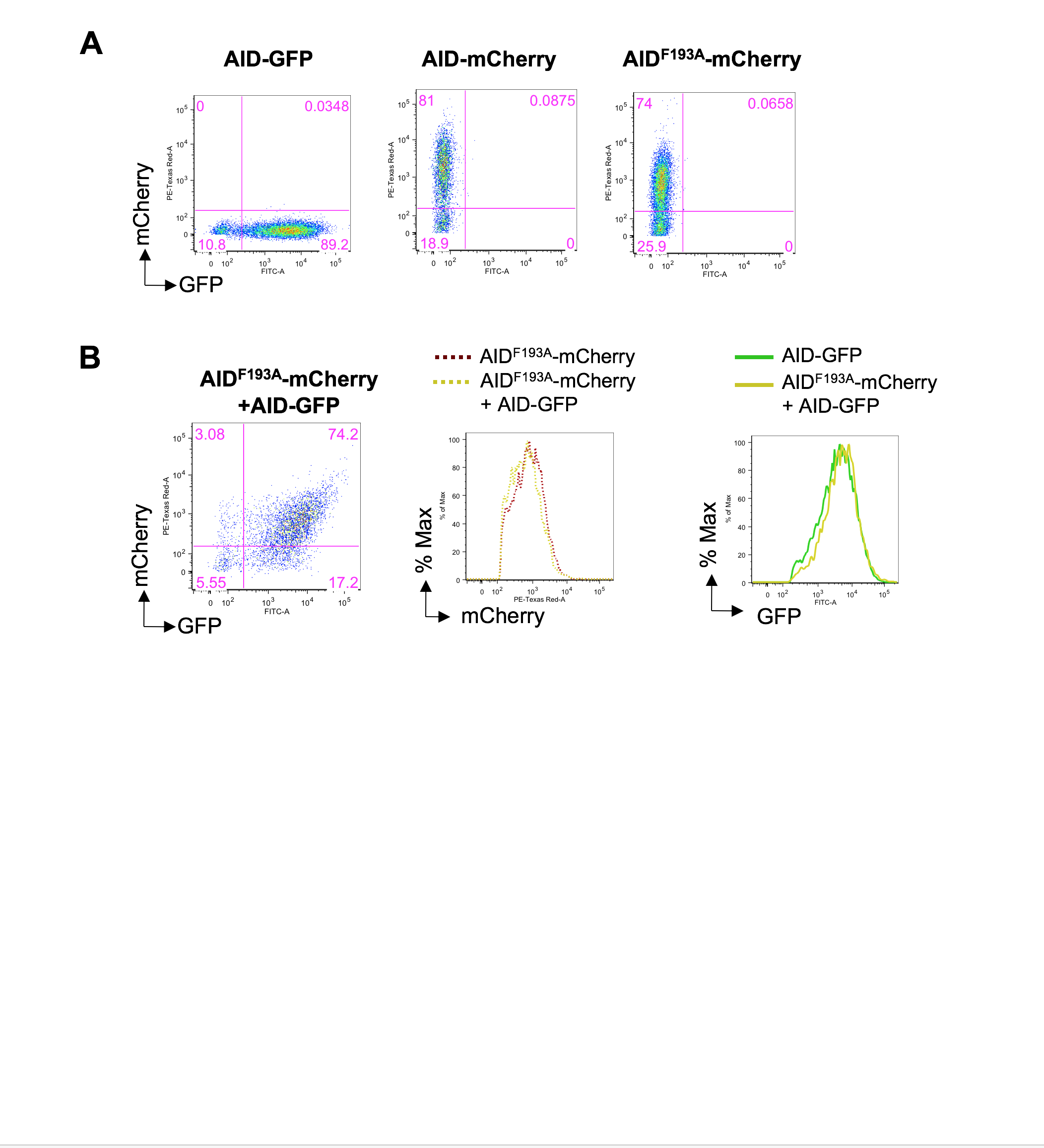

Supplement: S6 Fig — (A). Scatter plots of mCherry and GFP signals in HT1080 cells expressing indicated AID derivative(s). (B) Left, scatter plots of mCherry and GFP signals in HT1080 AID-GFP AIDF193A-mCherry double transfectants. Right, flow cytometry of indicated HT1080 transfectants, showing mCherry and GFP signals relative to maximum. (TIF) [file pgen.1007968.s007.tif]

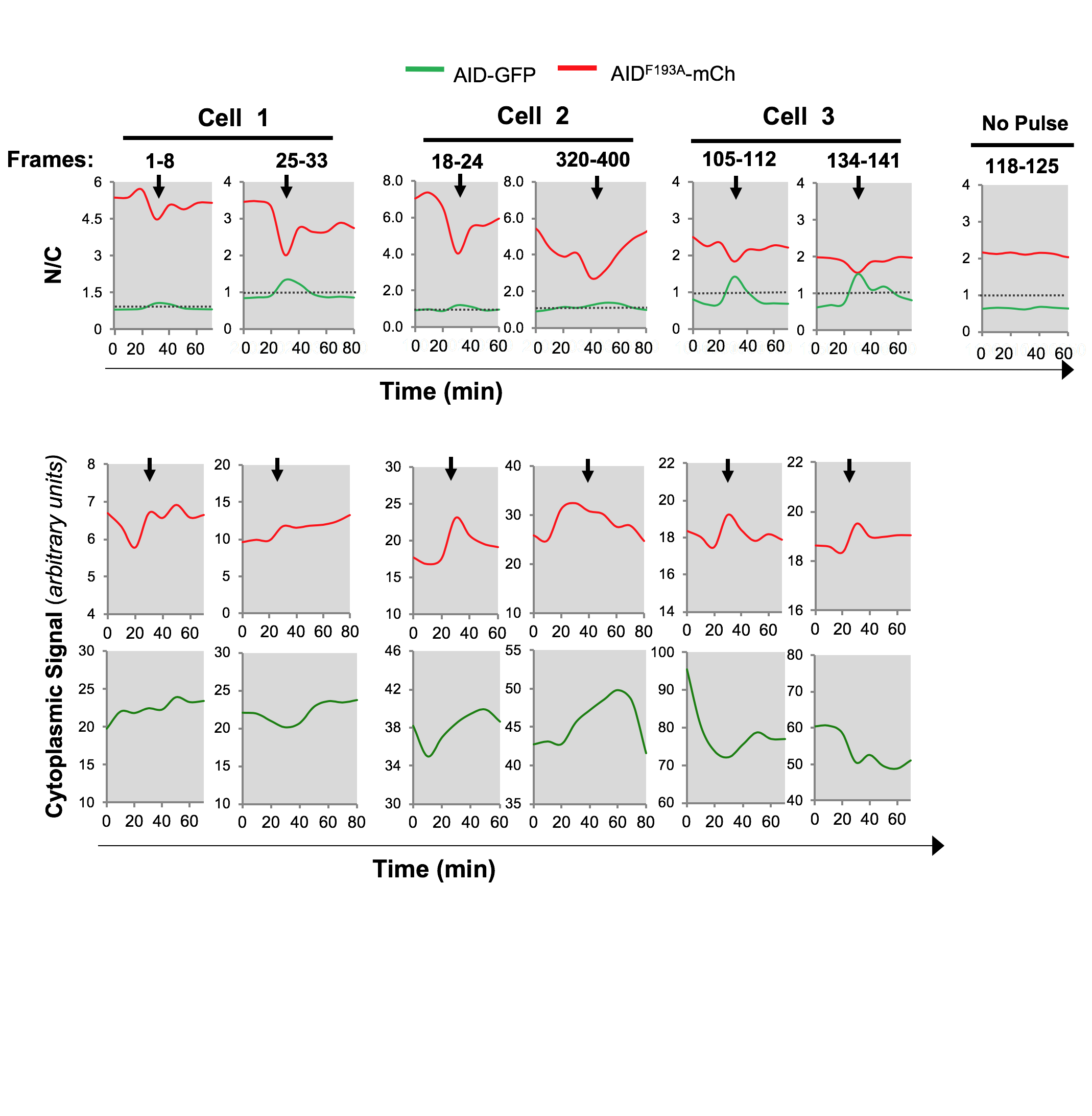

Supplement: S7 Fig — Above: Ratios of nuclear to cytoplasmic signals (N/C) for AID-GFP (green) and AIDF193A-mCherry (red) in two pulses and synchronous attenuation events spanning indicated frames for each of the three cells shown in Fig 4. Control quantification of the AID-GFP and AIDF193A-mCherry N/C ratio over a 60 min period when a cell was not pulsing yielded a relatively flat line, with frame-to-frame variations of <5% of total signal (far right). Arrows above tracings indicate times of peak N/C ratio for AID-GFP and of minimal N/C ratio for AIDF193A-mCherry signal; which correspond to peak of AIDF193A-mCherry cytoplasmic signal, above. Dotted line indicates nuclear/cytoplasmic signal ratio of one. Below: Cytoplasmic signal tracings for intervals corresponding to tracings of nuclear signals spanning indicated frames for each of the three cells shown in Fig 4. Arrows in panels in top row indicate times of peak AIDF193A-mCherry cytoplasmic signals. (TIF) [file pgen.1007968.s008.tif]
